# *Rhamnella brachycarpa* (Rhamnaceae), a new species from Hainan Island, China

**DOI:** 10.3897/phytokeys.132.36776

**Published:** 2019-09-26

**Authors:** Zhiqiang Lu, Yongshuai Sun

**Affiliations:** 1 CAS Key Laboratory of Tropical Forest Ecology, Xishuangbanna Tropical Botanical Garden, Chinese Academy of Sciences, Mengla 666303, Yunnan, China Xishuangbanna Tropical Botanical Garden, Chinese Academy of Sciences Mengla China

**Keywords:** *Rhamnella brachycarpa*, new species, Hainan Island

## Abstract

*Rhamnella brachycarpa* Z. Qiang Lu & Y. Shuai Sun, a new evergreen woody species from Hainan Island, is described and illustrated. The specimens of this new species have previously been identified and placed under *R. rubrinervis* (H. Lév.) Rehder, with which it shares evergreen leaves, erect and climbing habits and axillary flowering branches with bracteole leaves. However, the specimens from three distinct Hainan populations significantly differ from those of *R. rubrinervis* from other regions with smaller length to width ratios of leaves, fruit and seeds, smaller sizes of fruit and seeds and mucronate seed apices. Principal Component Analysis of the closely related taxa, based on multiple morphological characters, further recognised two separated groups. One of them comprises *R. tonkinensis* and *R. rubrinervis*, the other merely includes all individuals from these distinct Hainan populations. Therefore, *R. brachycarpa*, based on these distinct Hainan populations, is here erected as a new species, distinctly different from its published relatives.

## Introduction

**Figure 1.**
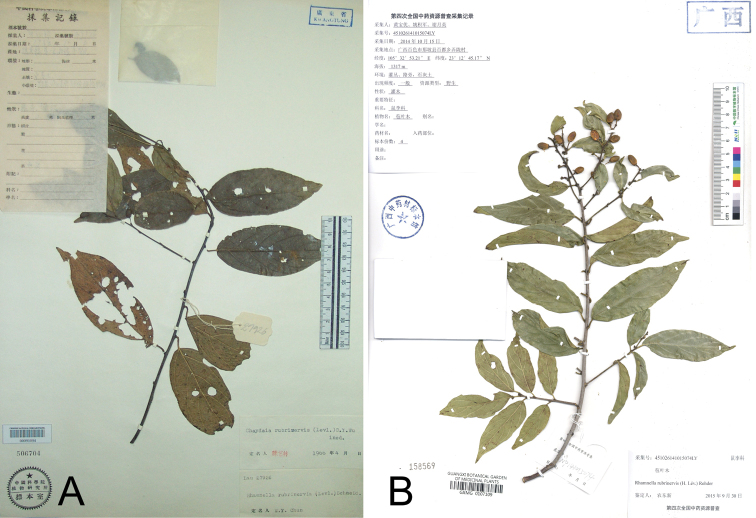
The gross morphology of two specimens identified as *Rhamnella rubrinervis*. **A***R. rubrinervis* from Hainan Island (Qionghai, Tayang, 27 Nov 1936, *X.Q. Liu 28256*, PE) **B***R. rubrinervis* from Guangxi (Baise, Napo, 15 Oct 2014, *B.Y. Huang et al. 074LY*, GXMG). The leaf length to width ratio and fruit size are totally different between two specimens.

The buckthorn family (Rhamnaceae) comprises 11 tribes and approximately 61 genera (Hauenschild et al. 2016). In this family, *Rhamnella* (Miquel, 1867) in the tribe Rhamneae is a small genus of shrubs, small trees and climbers (Chen and Carsten 2007; Hauenschild et al. 2016). In "Flora of China", *Rhamnella*, with eight described species, is recognised by the distinctly pedicellate flowers and fleshy fruits, 1-stoned drupes, pinnately veined leaves, serrate leaf margins, semi-inferior ovaries, stipules without thorns and flowers in axillary cymes (Chen and Carsten 2007). Most of them are deciduous broad-leaved woody species with erect habit. According to Flora of China, *R. rubrinervis* (H. Lév.) Rehder is the only evergreen species with both erect and climbing habits within this genus in China and is distributed in S and SE Yunnan, SW and S Guizhou, Guangxi and Hainan Island (Chen and Carsten 2007). However, four other closely related species, *R. tonkinensis* (Pit.) T. Yamaz., *R. hainanensis* Merr., *R. crenulata* (Hand.-Mazz.) T. Yamaz. and *R. longifolia* Tsai & K.M. Feng, have also been published (http://www.theplantlist.org). Only *R. rubrinervis* and *R. tonkinensis* have been accepted as valid species, while all others are treated as synonyms of *R. rubrinervis* (Chen and Carsten 2007). Interestingly, these evergreen species have been treated as an independent genus because of the unique morphology and *R. rubrinervis* has been proposed to be conspecific to *R. tonkinensis* (Fan and Yang 1997; Chen and Carsten 2007). After examining all specimens of *R. rubrinervis* preserved in the Chinese Virtual Herbarium (http://www.cvh.org.cn) and Global Biodiversity Information Facility (https://www.gbif.org/) in 2017, we found that one distinct specimen from Hainan Island placed under *R. rubrinervis* might represent a new species because it was clearly different from a representative specimen of this species from Guangxi with the characters of shorter fruit and smaller leaf length to width ratio (Figure 1). In order to further test this hypothesis, we re-examined specimens (including type specimens) in herbaria to illustrate the distinct differences of this special Hainan specimen. We also conducted a field survey across the total distribution range of *R. rubrinervis*, including this Hainan population, to collect enough specimens for the examination of their morphological variations at the population level.

## Material and methods

### Field surveys

We examined the specimens of *R. rubrinervis* preserved in the following herbaria: PE, KUN, GXMG, GXMI, GZAC, IBK, IMDY and MNHN (Table 1). In addition, we collected 164 specimens from 18 populations across its total distribution range for morphological comparison and clustering analysis. Voucher specimens were deposited as *Zhiqiang Lu 2018XTBG001–Zhiqiang Lu 2018XTBG019* (HITBC), *Zhiqiang Lu 2018191001–Zhiqiang Lu 2018191011* (HITBC), *Zhiqiang Lu 2018XSBN001–Zhiqiang Lu 2018XSBN003* (HITBC), *Zhiqiang Lu 2018115001–Zhiqiang Lu 2018115013* (HITBC), *Zhiqiang Lu 2018034001–Zhiqiang Lu 2018034007* (HITBC), *Zhiqiang Lu 2018027001–Zhiqiang Lu 2018027011* (HITBC), *Zhiqiang Lu 2018017001–Zhiqiang Lu 2018017008* (HITBC), *Zhiqiang Lu 2018128001–Zhiqiang Lu 2018128012* (HITBC), *Zhiqiang Lu 2018041001–Zhiqiang Lu 2018041007* (HITBC), *Zhiqiang Lu 2018033001–Zhiqiang Lu 2018033005* (HITBC), *Zhiqiang Lu 2018187001–Zhiqiang Lu 2018187003* (HITBC), *Zhiqiang Lu 2018141001–Zhiqiang Lu 2018141012* (HITBC), *Zhiqiang Lu 2018138001–Zhiqiang Lu 2018138015* (HITBC), *Zhiqiang Lu 2018035001–Zhiqiang Lu 2018035006* (HITBC), *Zhiqiang Lu 2018106001–Zhiqiang Lu 2018106009* (HITBC), *Zhiqiang Lu 2018017001–Zhiqiang Lu 2018017008* (HITBC), *Zhiqiang Lu 2018HN3001–Zhiqiang Lu 2018HN3012* (HITBC) and *Zhiqiang Lu 2018HN3013–Zhiqiang Lu 2018HN3015* (GXMI). In addition, we specifically visited the site of this suspected new species and conducted specific field surveys on its total distribution range and a population census on Hainan Island, from 2018 to 2019.

### Morphological analysis

The newly collected specimens of *R. rubrinervis* were used to conduct morphological comparisons, based on the characters of leaves, flowers, fruit and seeds. We examined their morphological variations within and between populations through the measurement of the typical leaf and fruit for each of 164 newly collected specimens. Seventy five specimens preserved in herbaria were also used to conduct the morphological measurement and comparison (Table 1). However, the measurement of seed characters for these specimens was abandoned, because sizes of seeds could also be reflected by those of fruit (Table 2), in addition that, we could not avoid the damage to them during this process. Finally, a total of 239 specimens and 10 morphological characters were used to carry out the Principal Component Analysis (PCA) (Table 3).

**Table 1.** Specimens preserved in herbarium used for Principal Component Analysis (PCA) of morphological variations. Those collection sites marked in bold indicate where the additional specimens were collected in this study.

Species name	Collector	Collection number	Collection site	Herbarium	No. of specimen
*R. brachycarpa*	*Z.Q. Lu*	*2018HN3001–2018HN3012*	**Baoting, Hainan**	XTBG	12
	*Z.Q. Lu*	*2018HN3013–2018HN3015*	**Baoting, Hainan**	GXMI	3
	*X.Q. Liu*	*28256*	Qionghai, Hainan	PE	1
*R. hainanensis*	*F.A. McClure*	*8358* (*three copies*)	Wuzhishan, Hainan	CAS	3 (isotypes)
*R. rubrinervis*	*Z. Huang*	*34582*	Sanya, Hainan	PE	1
	*Tsang and Fung*	*18207*	Danzhou, Hainan	PE	1
*R. tonkinensis*	*H.F. Bon*	*2246* (*three copies*)	Vietnam	MNHN	3 (isolectotypes)
*R. longifolia*	*G.M. Feng*	11638	Jinghong, Yunnan	KUN	1 (isotype)
*R. crenulata*	*Handel-Mazzetti*	*10758*	Badschai, Guizhou	HT	1 (isotype)
*R. rubrinervis*	*A. Rehder*	*729*	China	HUH	1 (holotype)
	*China-Vietnam team*	*1477* (*two copies*)	N Vietnam	PE, KUN	2
	*Q.W. Wang*	*76350*, *75597*, *75473*, *441*	**Jinghong, Yunnan**	PE	4
	*G.M. Feng*	*11638*, *14381*, *159*	Menghai, Yunnan	PE	3
	*Y.H. Li*	*3574*, *34298*, *3574*, *2505*	**Menglun, Yunnan**	HITBC	4
	*G.D. Tao*	*16289*	**Jinuoshan, Yunnan**	HITBC	1
	*X.W. Li*	*13103*, *13015*	Yiwu, Yunnan	KUN	2
	*Z.H. Yang*	*1424*	**Fadou, Yunnan**	KUN	1
	*Maguan team*	*076*, *323*	Maguan, Yunnan	IMDY	2
	*Y.Z. Wang et al.*	*4388*	**Malipo, Yunnan**	PE	1
	*Q.W. Wang*	*84943*, *84942*	Yanshan, Yunnan	PE	2
	*S.W. Yu*	*86011*, *860117*	Yanshan, Yunnan	KUN	2
	*Beijing team*	*893045*, *896924*	**Tian’e, Guangxi**	PE	2
	*S.X. Yu*	*337*	**Napo, Guangxi**	GXMG	1
	*Anonymous*	*63*, *402*	Location unknown	KUN	2
	*China-Japan team*	*100828*	**Xingyi, Guizhou**	KUN	1
	*Guizhou team*	*8180*, *8427*	Xingren, Guizhou	KUN	2
	*J.C. Yang*	*LH0072*	Longtan, Guangxi	IBK	1
	*Anonymous*	*683*	**Liuzhou, Guangxi**	IBK	1
	*Lingyun team*	*19LY*, *08LY*, *48LY*, *49LY*, *58LY*, *74LY*, *51LY*, *04LY*, *05*	**Lingyun, Guangxi**	GXMG	9
	*Tianlin team*	*031* (*two copies*), *005*	**Tianlin, Guangxi**	GXMG	3
	*Yongfu team*	*03LY*	Yongfu, Guangxi	GXMG	1
	*Anonymous*	*00747*, *99LY*	**Tian’e, Guangxi**	IBK, GXMG	2
	*B.Y. Huang*	*23672*	Shangsi, Guangxi	GXMG	1
	*C.C. Huang*	*16994*, *00938*	Luocheng, Guangxi	GXMI	2
	*X.X. Chen*	*02793*, *02323*, *2967*	**Chongzuo, Guangxi**	GXMI	3
	*Y.S. Huang et al.*	*LYJX0657*, *LYJX0458*	**Jingxi, Guangxi**	IBK	2
	*K.M. Lan*	*870007*, *870060*	Daozhen, Guizhou	GZAC	2
	*R.C. Peng*	*ML0367*	Huanjiang, Guangxi	IBK	1
	*Z.Y. Cao*	*1135*, *597*	**Ceheng, Guizhou**	PE	2
	*Y. Jiang*	*7045*	**Dushan, Guizhou**	PE	1

**Table 2.** Morphological characters of *Rhamnella rubrinervis* and *R. brachycarpa* at the population level. Traits that differ between species are marked in bold.

Characters	*Rhamnella rubrinervis*	*Rhamnella brachycarpa*
**LEAF**		
Shape and size	Leaf blade oblong or ovate-oblong, 5.4–14.4 × 1.7–5.1 cm, **length to width ratio (2.5)2.8–3.9**; base commonly rounded, rarely cuneate, margin inconspicuously remotely serrate or subentire; **apex acuminate to long acuminate**; bracteole leaf similar to leaves in vegetative branches, but smaller	Leaf blade elliptic-ovate, 5.8–10.3 × 3.1–4.8 cm, **length to width ratio 1.9–2.4**; base cuneate or nearly rounded, margin inconspicuously remotely serrate or subentire; **apex short acuminate or acute**; bracteole leaf similar to leaves in vegetative branches, but smaller
**Length of petiole**	**3–9 mm**	**7–12 mm**
Number of lateral veins on each side of midvein	5–8	5–7
Average distance between lateral veins located in the middle of leaf	3–8 mm	3–6 mm
**FLOWER**		
Number of flowers for each axillary cyme	2–10	2–9
Length of pedicel	2–5	3–5
Shape and size	Flower diameter ca. 4 mm; sepals triangular, ca. 2 mm; stamens involute by petals, ca. 2 mm in length	Flower diameter ca. 4 mm; sepals triangular, ca. 2 mm; stamens involute by petals, ca. 2 mm in length
**FRUIT**		
**Size of fleshy fruit**	**10.2–12.1 × 10.1–12.5 mm**	**8.7–10.9 × 7.5–10.6 mm**
**Size of dried fruit**	**8.2–11.1 × 4.2–5.8 mm**	**6.5–7.5 × 4.7–6.0 mm**
**Length to width ratio of dried fruit**	**1.6–2.2**	**1.3–1.5**
Length of fruiting pedicel	3–6 mm	4–6 mm
**SEED**		
**Size of seed**	**7.1–9.9 × 4.0–5.5 mm**	**5.0–7.0 × 4.5–5.5 mm**
**Length to width ratio of seed**	**1.6**–**2.1**	**0.9**–**1.5**
**Seed apex**	**Rarely mucronate**	**Mucronate**

## Results

**Figure 2.**
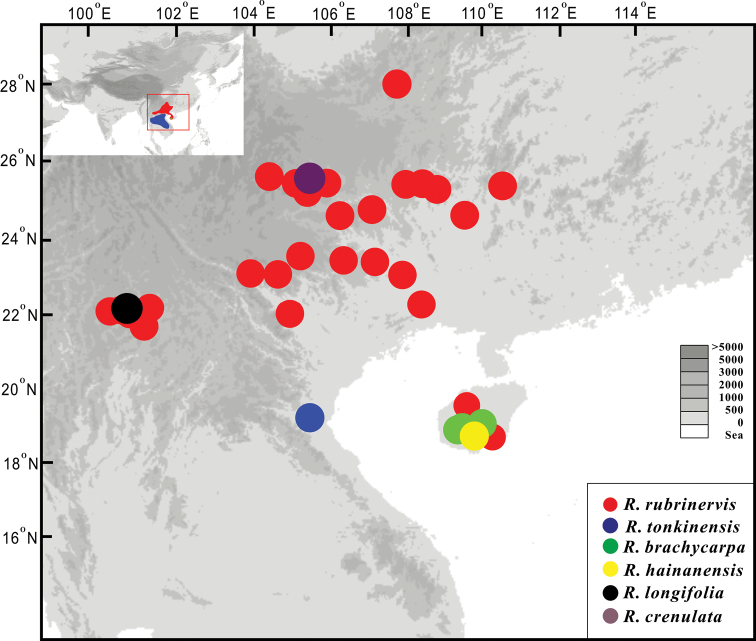
Distribution and locations of *R. brachycarpa*, *R. rubrinervis* and *R. tonkinensis*, based on field surveys, Chinese Virtual Herbarium (http://www.cvh.org.cn/) and Global Biodiversity Information Facility (https://www.gbif.org/).

**Figure 3.**
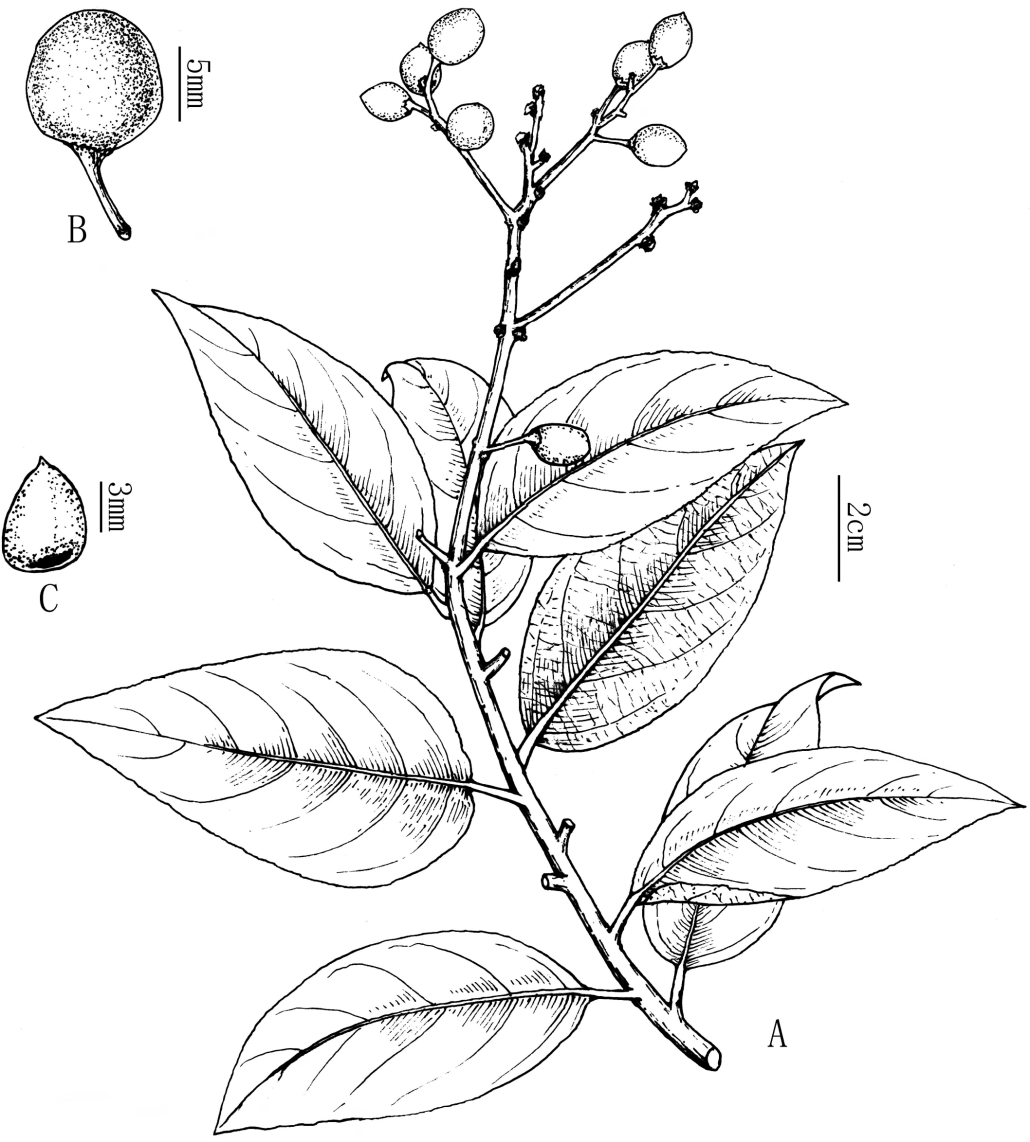
*Rhamnella brachycarpa* Z. Qiang Lu & Y. Shuai Sun, sp. nov., drawn from the tree of *Z.Q. Lu 2018HN3001*.

**Figure 4.**
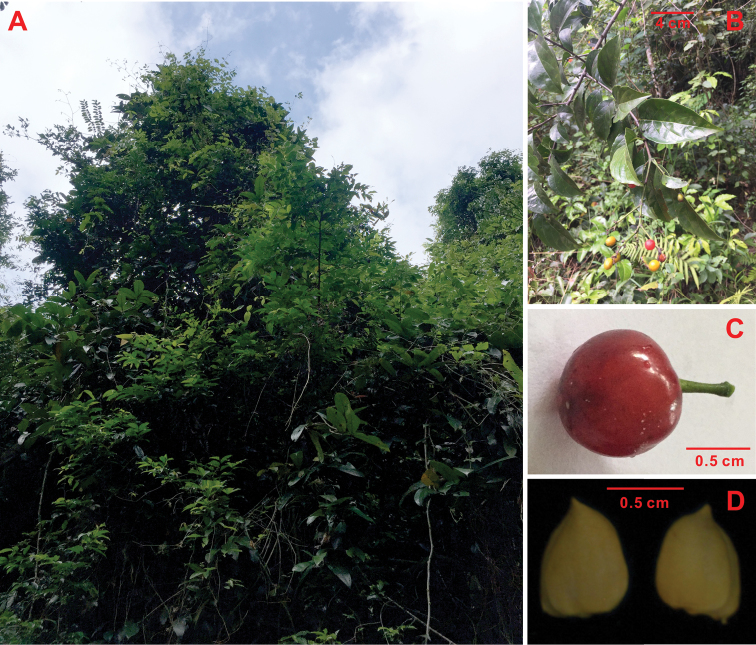
*Rhamnella brachycarpa* Z. Qiang Lu & Y. Shuai Sun. **A** The habitat **B** Branches with leaves and fruit **C** Fruit **D** Seeds.

**Figure 5.**
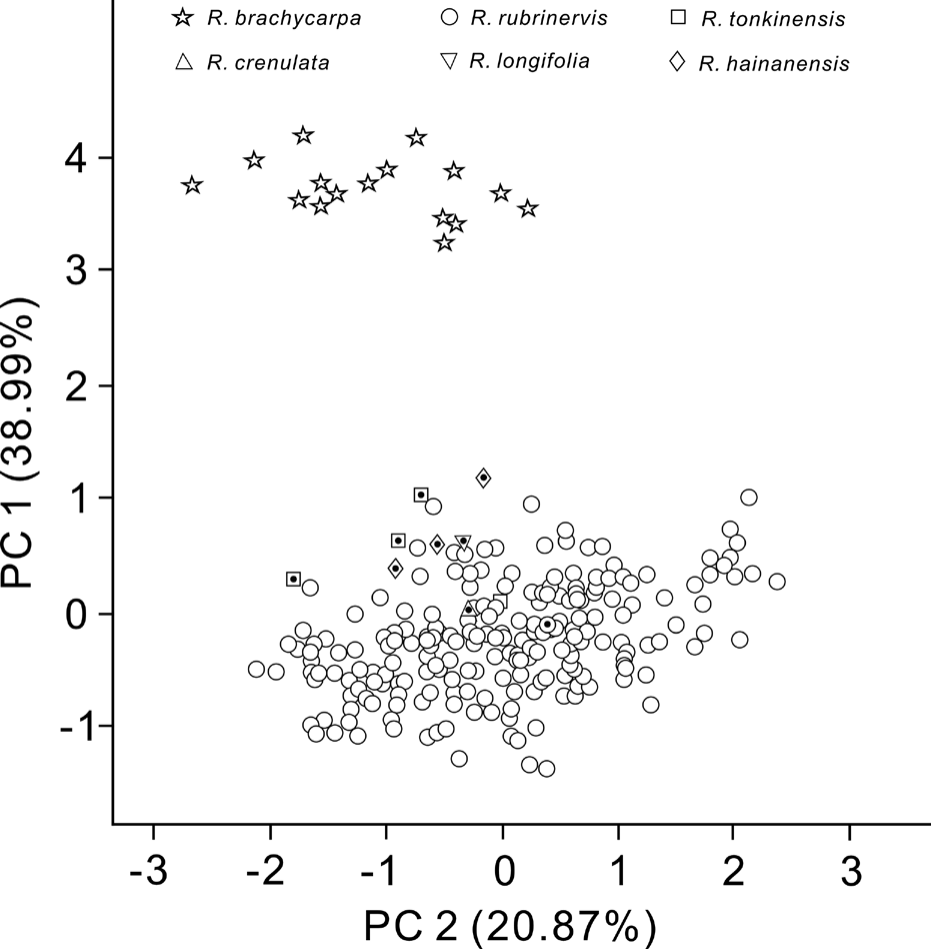
Morphological clustering based on Principal Component Analysis. *R. crenulata*, *R. longifolia* and *R. hainanensis* are synonyms of *R. rubrinervis*. The black dots indicate the designated type specimens.

Our specific field survey on the special record on Hainan Island showed that no extant tree similar to *R. rubrinervis* existed in Tayang Township, Qionghai City (Figure 1A) and that the habitat was badly destroyed by human activity. However, we finally explored two new *R. rubrinervis* populations with smaller leaf length to width ratio and shorter fruit in Baoting County during our field surveys in 2018 (Table 1; Figures 2–4). Morphological comparison at the population level was conducted, showing three Hainan populations distinctly differed from those of *R. rubrinervis* from other regions with the smaller leaf length to width ratio (1.9–2.4 vs. (2.5) 2.8–3.9), shorter fruit (6.5–7.5 mm vs. 8.2–11.1 mm), smaller fruit length to width ratio (1.3–1.5 vs. 1.6–2.2), dumpier seeds (5.0–7.0 × 4.5–5.5 mm vs. 7.1–9.9 × 4.0–5.5 mm), smaller seed length to width ratio (0.9–1.5 vs. 1.6–2.1) and mucronate seed apices (Figures 3–4; Table 2). Differences were also found in leaf base shape, petiole length (3–9 mm vs. 7–12 mm) and fleshy fruit size (8.7–10.9 × 7.5–10.6 mm vs. 10.2–12.1 × 10.1–12.5 mm). No significant difference in flowers was found between the distinct Hainan populations and those of *R. rubrinervis* from other regions. A further PCA of all closely related specimens (including historical type specimens of four published synonyms), based on 10 morphological characters, was carried out (Table 3), distinguishing these three Hainan populations (in Baoting and Qionghai) and those of *R. tonkinensis* and *R. rubrinervis* from other regions into two major groups (Figure 5). One of the two groups merely represented these distinct Hainan populations; the remaining populations of *R. tonkinensis* and *R. rubrinervis* formed the other group. The first principal component axis (PC1; accounting for 38.99% of the variation) significantly separated these distinct Hainan populations from the other two species, while the second principal component axis (PC2; accounting for 20.87% of the variation) failed in separating both groups (Figure 5).

**Table 3.** Morphological characters measured for Principal Component Analysis (PCA) based on 239 specimens.

Character number	State	Unit	Coding (if qualitative)
**LEAF**			
1	Length	cm	
2	Width	cm	
3	Length to width ratio	Ratio	
4	Length of petiole	cm	
5	Apex	Qualitative	2 = Short acuminate or acute
			1 = Long acuminate or acuminate
6	Base rounded or cuneate	Qualitative	2 = Cuneate
			1 = Rounded
7	Number of lateral veins on each side of midvein	Count	
**DRIED FRUIT**			
8	Length	mm	
9	Width	mm	
10	Length to width ratio	Ratio	

## Discussion

In this study, we demonstrated that three distinct Hainan populations previously placed under *R. rubrinervis* should be described as a new species, based on the distinct morphology and clustering analysis at the population level. Morphological comparison showed that they differ from those of *R. rubrinervis* from other regions in mucronate seed apices, shorter fruit, dumpier seeds and smaller length to width ratios of leaves, fruit and seeds. PCA analysis further clustered these special Hainan populations into a separated group distinctly different from those of *R. rubrinervis* and *R. tonkinensis* from other regions (including historical type specimens of those published synonyms). Our results also showed that *R. rubrinervis* and *R. tonkinensis* had a similar morphology, indicating the obscure species boundary between them. This finding is consistent with a previous study (Fan and Yang 1997). Obviously, these special Hainan populations are distinctly different from all extant relatives from other regions (Chen and Carsten 2007). Given this, we describe them in the following as a new species.

## Taxonomic treatment

### Rhamnella brachycarpa

Taxon classificationPlantaeRosalesRhamnaceae

Z. Qiang Lu & Y. Shuai Sun sp. nov.

2FBD1E68-671D-5A5B-A400-8F82A0ADD764

urn:lsid:ipni.org:names:60479375-2

Figures 3 , 4

#### Diagnosis.

*Rhamnella brachycarpa* differs from *R. rubrinervis* and *R. tonkinensis* by leaf length to width ratio of 1.9–2.4 (compared to 2.5–3.9 in the related species) and dried fruit 6.5–7.5 × 4.7–6.0 mm in size (compared to 8.2–11.1 × 4.2–5.8 mm in *R. rubrinervis* and *R. tonkinensis*) with length to width ratio of 1.3–1.5 (compared to 1.6–2.2).

#### Type.

CHINA. Hainan: Baoting County, Xian’an, 109°25'34"E, 18°35'37"N, 650 m alt., forest edge, 16 Oct 2018, *Z.Q. Lu 2018HN3001* (holotype, HITBC; isotypes, HITBC and GXMG).

#### Description.

Small trees or climbing vines, evergreen. Young branches sparsely pilose or glabrous; older branches reddish-brown, grey-brown or grey, glabrous. Leaves alternate; stipules lanceolate, persistent; petiole 0.7–1.2 cm long, glabrous, narrowly grooved on the upper surface; leaf blade abaxially dark green, shiny, adaxially pale green, elliptic-ovate, 5.8–10.3 × 3.1–4.8 cm, leathery, abaxially glabrous, adaxially glabrous, lateral veins 5–7 pairs, slightly impressed abaxially, prominent adaxially, base cuneate or nearly rounded, margin subentire, conspicuously serrate when seedlings, apex shortly acuminate or acute. Flowering branches axillary, rarely not, 7–18 cm long, glabrous. Flowers bisexual, ca. 4 mm diam., 4– or 6–merous, few to 2 or 9 in axillary cymes, subsessile or shortly pedunculate at bracteole leaf of flowering branches; bracteole leaf similar to leaves in vegetative branches, but smaller, 1.5–5.0 × 0.9–2.3 cm. Pedicel 3.3–5.2 mm long, glabrous. Sepals triangular, ca. 2 mm, adaxially midvein raised, rostellate at lower middle. Petals obovate, shortly clawed. Stamens involute by petals, ca. 2 mm long. Disc rounded, thick. Ovary globose, not immersed in disc. Drupe purple-red or orange at maturity, ovoid-cylindrical or globose, 8.7–10.9 × 7.5–10.6 mm, 6.5–7.5 × 4.7–6.0 mm when dried, base with persistent calyx tube; fruiting pedicel 4.3–6.2 mm, glabrous, 1-loculed, 1-seeded; seed dumpy, apex mucronate, smooth on the surface, 5.0–7.0 × 4.5–5.5 mm.

#### Etymology.

In contrast with relatives, all individuals from these distinct Hainan populations have shorter fruit; we therefore give the epithet *Rhamnella brachycarpa*.

#### Phenology.

Flowering from May to September and fruiting from July to October.

#### Habitat, distribution and conservation.

According to our field surveys and records in Chinese Virtual Herbarium (CVH), *R. brachycarpa* has been found at three sites on Hainan Island (Baoting County: Xian’an and Shijia; Qionghai City: Tayang). Only two small populations with 45 individuals (including only 22 mature trees) have been found in Baoting, while no extant tree has been found in the Qionghai population due to the destruction of the habitat. Of the two small populations in Baoting, with a separation distance of about 900 m, one consists of only three mature trees without seedlings; the second population has 19 mature trees and 23 immature trees. All mature trees are more than 3.1 m in height and less than 1.8 m is observed for all immature trees. Forty five individuals of this new species sparsely grow along the roadsides and in forest edges or thick forest. Mature trees with climbing habit only grow in thick forest or forest edges with high canopies, but those with erect habit prefer to grow along roadsides or in forest edges without high canopies. Nevertheless, all these immature trees, sparsely growing along the roadsides and in forest edges or thick forest, present the erect habit. The rarity of this new species may be partially due to human activity because the habitat is also suitable for cultivating rubber trees and other economic plants. Comparing to the number of mature trees, the number of immature trees further indicates a decreasing population trend. In addition, all these trees are distributed in a total area of approximately 0.7 km^2^. No population was found during repeated field surveys of the surrounding areas. According to the IUCN Categories and Criteria (IUCN 2016), the species is classified as “Critically Endangered” (CR). Therefore, it will be necessary to pay close attention to the conservation of this new species.

#### Additional specimens examined.

**CHINA. Hainan**: Qionghai City, Tayang, open forest, 27 Nov 1936, *X.Q. Liu 28256* (PE); Baoting County, Xian’an, 109°25'34"E, 18°35'37"N, 650 m alt., forest edge, roadside and thick forest, 16 Oct 2018, *Z.Q. Lu 2018HN3002*–*Z.Q. Lu 2018HN3012* (HITBC); Baoting County, Shijia, 109°25'42"E, 18°36'02"N, 680 m alt., forest edge, 17 Oct 2018, *Z.Q. Lu 2018HN3013*–*Z.Q. Lu 2018HN3015* (GXMI).

## Supplementary Material

XML Treatment for Rhamnella brachycarpa
